# Membrane biofilm development improves COD removal in anaerobic membrane bioreactor wastewater treatment

**DOI:** 10.1111/1751-7915.12311

**Published:** 2015-08-04

**Authors:** Adam L Smith, Steven J Skerlos, Lutgarde Raskin

**Affiliations:** 1 Department of Civil and Environmental Engineering, University of Michigan2350 Hayward Road, Ann Arbor, MI, 48109, USA; 2 Department of Mechanical Engineering, University of Michigan2350 Hayward Road, Ann Arbor, MI, 48109, USA

## Abstract

Membrane biofilm development was evaluated to improve psychrophilic (15°C) anaerobic membrane bioreactor (AnMBR) treatment of domestic wastewater. An AnMBR containing three replicate submerged membrane housings with separate permeate collection was operated at three levels of membrane fouling by independently controlling biogas sparging for each membrane unit. High membrane fouling significantly improved permeate quality, but resulted in dissolved methane in the permeate at a concentration two to three times the equilibrium concentration predicted by Henry’s law. Illumina sequencing of 16S rRNA targeting *Bacteria* and *Archaea* and reverse transcription-quantitative polymerase chain reaction targeting the methyl coenzyme-M reductase (*mcrA*) gene in methanogens indicated that the membrane biofilm was enriched in highly active methanogens and syntrophic bacteria. Restoring fouled membranes to a transmembrane pressure (TMP) near zero by increasing biogas sparging did not disrupt the biofilm’s treatment performance, suggesting that microbes in the foulant layer were tightly adhered and did not significantly contribute to TMP. Dissolved methane oversaturation persisted without high TMP, implying that methanogenesis in the biofilm, rather than high TMP, was the primary driving force in methane oversaturation. The results describe an attractive operational strategy to improve treatment performance in low-temperature AnMBR by supporting syntrophy and methanogenesis in the membrane biofilm through controlled membrane fouling.

## Introduction

Anaerobic membrane bioreactor (AnMBR) treatment allows for the direct recovery of energy from wastewater in the form of methane-rich biogas. In AnMBRs, methane is produced during the anaerobic microbial degradation of the organic compounds present in wastewater in a bioreactor containing microbial biomass in suspension. This suspended biomass is separated from the treated wastewater using membrane filtration to produce a particle-free wastewater effluent (permeate). The recent recognition of the potential benefits of AnMBR treatment of domestic wastewater compared with conventional activated sludge treatment has resulted in a surge in AnMBR research activity (e.g. Yoo *et al*., 2012; Ma *et al*., 2013; Smith *et al*., 2015) and pilot-scale evaluations (Dagnew *et al*., 2011; Gimenez *et al*., 2011; Martinez-Sosa *et al*., 2011; Robles *et al*., 2013; Shin *et al*., 2014; Gouveia *et al*., 2015). As the pumping energy demand needed for membrane filtration increases during the development of a membrane fouling layer, membrane fouling has received considerable attention in AnMBR research (Gao *et al*., 2010; Huang *et al*., 2011; Yang *et al*., 2011; Chen *et al*., 2012; Kola *et al*., 2014). The consensus in the water quality engineering field has been to operate membrane-filtration systems, including AnMBRs, with minimal membrane fouling (Yang *et al*., 2006), which is accomplished using gas sparging, backflushing and chemical cleaning. As a result, almost no research has been performed on the potential benefits of membrane fouling. The membrane fouling layer contains considerable microbial biomass and can thus be considered a membrane biofilm, which has the potential to improve effluent quality by providing additional biodegradation not accomplished by the suspended biomass (Smith *et al*., 2015).

Anaerobic microbial communities in a membrane biofilm could have an advantage over suspended microbial communities because of reduced mass-transfer limitations. Mass-transfer phenomena likely have a substantial effect on substrate utilization when substrate concentrations are low, such as during domestic wastewater treatment (Gonzalez-Gil *et al*., 2001), at low temperatures (Wu *et al*., 1995) and when mass transport is influenced by advective forces such as liquid flow through a membrane biofilm. In addition, biofilms may facilitate interspecies hydrogen transfer (Ishii *et al*., 2005; 2006) or direct interspecies electron transfer (DIET; Summers *et al*., 2010; Morita *et al*., 2011) between methanogens and their syntrophic partners, and thus provide enhanced microbial activity relative to the suspended biomass activity.

The complexity of anaerobic microbial communities and reported differences in suspended and biofilm AnMBR community structure (Gao *et al*., 2010; Yu *et al*., 2012; Ma *et al*., 2013; Smith *et al*., 2013; 2015) suggest that careful monitoring of community structure during development of AnMBR operational strategies is important. RNA-based approaches targeting either 16S rRNA (e.g. Eichler *et al*., 2006; Foesel *et al*., 2013; Hunt *et al*., 2013; Männistö *et al*., 2013) or transcripts of functional genes [e.g. the methyl coenzyme-M reductase (*mcrA*) gene in methanogens; Freitag and Prosser, 2009] may be more useful than DNA-based approaches in characterizing microbial community function in AnMBRs. The need for RNA-based approaches is particularly important given the slow growth rates and low biomass yields of anaerobic microbes, high biomass retention provided by membrane separation, and short operational periods commonly studied in AnMBRs especially relative to the long solids retention time (SRT) in these systems.

This study elucidated the contribution of the membrane biofilm in AnMBR treatment of synthetic domestic wastewater using a bench-scale AnMBR operated at 15°C equipped with three submerged membrane housings, designated P1, P2 and P3, with separate permeate collection and independent biogas sparging control. The three membrane housings were operated to allow for three different levels of membrane fouling and membrane biofilm development. Illumina sequencing of 16S rRNA genes (rDNA) and 16S rRNA and reverse transcription-quantitative polymerase chain reaction (RT-qPCR) targeting the *mcrA* gene transcripts were applied to compare microbial community structure and activity dynamics in the suspended biomass and in the membrane biofilms.

## Results and discussion

### Slow start-up after inoculating the psychrophilic AnMBR with mesophilic sludge

**Figure 1:**
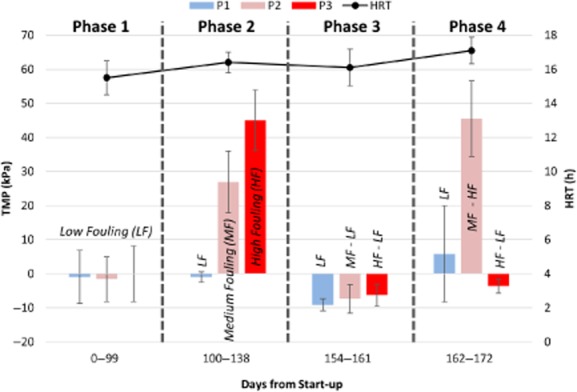
Average transmembrane pressure (TMP) for each of the membranes P1, P2 and P3 (left y-axis) and bioreactor hydraulic retention time (HRT; right y-axis) from days 0 to 172. This time period is divided in four phases defined by the degree of membrane fouling or biofilm development. Data from days 139 to 153 are not reported due to poor AnMBR performance. Error bars for HRT represent the standard deviation of daily flow rate measurements. Error bars for TMP represent the standard deviation of pressure data recorded every minute of operation.

The AnMBR with three membrane housings was initially operated for 99 days (Phase 1; Fig. 1) under low fouling (LF) conditions by maintaining a high biogas sparging flow rate to prevent biofilm development for each of the membrane units. COD removal during the first 99 days of operation (Phase 1) was limited, averaging 57% ± 12% (Fig. S1). The majority of the permeate chemical oxygen demand (COD) was comprised of acetate (average 70 ± 19 mg l^−1^) and propionate (average 52 ± 18 mg l^−1^) (Fig. S2). Further information regarding start-up is presented in Appendix S6.

### Biofilm development improves effluent quality but results in dissolved methane oversaturation

To improve permeate quality, a controlled membrane fouling experiment was conducted to encourage biofilm development on P2 and P3 by independently reducing the biogas sparging flow rates (Phase 2). Three different levels of membrane fouling were targeted – low fouling (LF; P1), medium fouling (MF; P2) and high fouling (HF; P3) – resulting in the need to operate with different transmembrane pressures (TMPs) to maintain similar fluxes. During Phase 2, P1, P2 and P3 TMP averaged −0.96 ± 1.5, 27 ± 9.0 and 45 ± 8.9 kPa, respectively, indicating the targeted fouling levels were achieved (Fig. 1). Hereafter, P1, P2 and P3 are referred to based on their fouling level (LF, MF and HF respectively).

**Figure 2:**
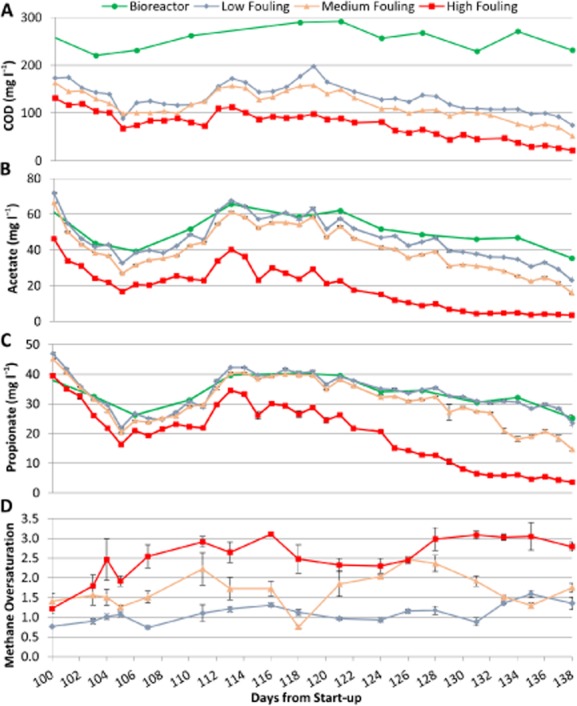
Effect of different degrees of biofilm development (low fouling, medium fouling and high fouling) on permeate quality during Phase 2 of AnMBR operation.A. Bioreactor (soluble) and permeate COD concentrations. Influent COD was 410 ± 46 mg l^−1^.B. Bioreactor and permeate acetate concentrations. Error bars represent the standard deviation of triplicate IC injections.C. Bioreactor and permeate propionate concentrations. Error bars represent the standard deviation of triplicate IC injections.D. Dissolved methane oversaturation in the permeate calculated assuming a Henry’s law constant of 34 300 atm at 15°C (Tchobanoglous *et al*., 2003), and measured methane partial pressure in the biogas and dissolved methane concentration in the permeate. The methane content of the biogas was approximately 90%, with the balance being carbon dioxide. A high methane content is expected given the low organic loading rate and differences in methane and carbon dioxide solubility at this temperature. Error bars represent the standard deviation of duplicate dissolved methane extractions and triplicate GC injections of each dissolved methane extract.

Differences in permeate COD concentrations were observed throughout Phase 2 and corresponded to the level of membrane fouling (Fig. 2A). The HF permeate consistently had the lowest COD with a concentration of 22 mg l^−1^ at the end of Phase 2. Permeate volatile fatty acid (VFA) levels showed a similar trend (Fig. 2B and C). The VFA concentrations in the bioreactor and LF permeate were similar throughout Phase 2, indicating minimal biological activity across the LF membrane. These observations indicate that controlled membrane fouling can substantially improve effluent quality in AnMBR, and further suggest that the activity of syntrophic propionate oxidizing populations and their methanogenic partners can be promoted through membrane biofilm development (see below).

Consistent with this, dissolved methane concentrations in MF and HF permeates indicated significant oversaturation of methane (Fig. 2D), suggesting that methanogenesis occurred in the biofilm and that methane produced in the biofilm left the system in the dissolved form. From days 107 to 138, dissolved methane concentrations in LF, MF and HF permeate averaged 1.1 ± 0.22, 1.7 ± 0.44 and 2.6 ± 0.30 times the concentrations predicted by Henry’s law respectively. The dissolved methane concentration in the bioreactor, which could not be measured, was believed to be near saturation due to vigorous biogas sparging. The observation that the dissolved methane concentration in LF permeate was close to saturation during Phase 2, as it was during Phase 1 for all three permeates, provided further evidence of minimal biological activity during LF conditions. Dissolved methane recovery downstream of anaerobic treatment has been attempted using membrane degasification, but substantial energy was required (Bandara *et al*., 2011) and methane content of the off-gas may be insufficient for cogeneration (Cookney *et al*., 2012). If released to the atmosphere, this ‘lost’ energy source could constitute a potent greenhouse gas emission (Smith *et al*., 2014). Biological oxidation of dissolved methane using aerobic methanotrophy or nitrite-dependent anaerobic methane oxidation (n-damo) could prevent greenhouse gas emissions but would require an additional unit process and energy input and would not recover the methane for electricity production.

### Biofilm development leads to a specialized microbial community enriched in active methanogens

**Figure 3:**
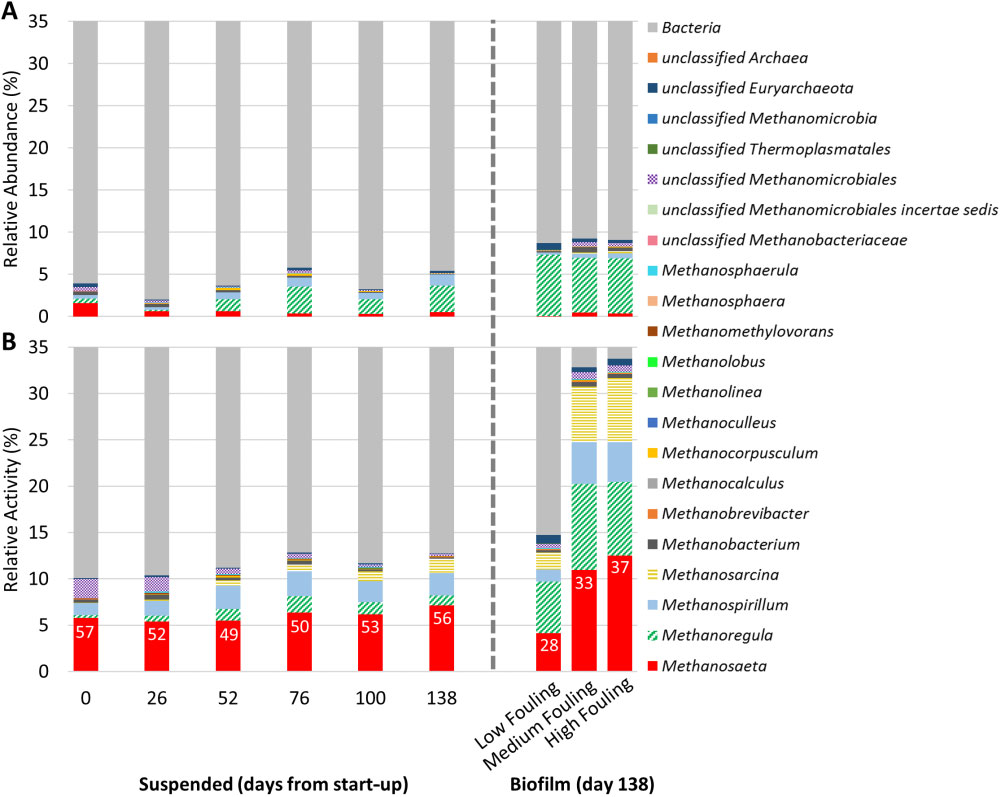
(A) Relative abundance of methanogens identified at the genus level based on 16S rDNA sequencing and (B) relative activity of methanogens identified at the genus level based on 16S rRNA sequencing. Data were obtained for suspended biomass samples collected from start-up to the end of Phase 2 and in biofilm samples obtained at the end of Phase 2. Data are expressed as percentages and were normalized using the total number of 16S rDNA sequences (A) and 16S rRNA sequences (B) (including *Bacteria* and *Archaea*). Numbers within bars in (B) represent the relative activity of *Methanosaeta* spp. based on 16S rRNA sequence data normalized to the 16S rRNA sequences of all *Archaea*. A truncated y-axis (0–35%) is shown to accentuate changes in methanogen abundance and activity.

High-throughput sequencing of both 16S rDNA and 16S rRNA was employed to study controlled membrane fouling during Phase 2, and the terms ‘relative abundance’ and ‘relative activity’, respectively, are used to report the results of these sequencing efforts. The relative abundance and relative activity levels of populations differed greatly in suspended and biofilm biomass (Fig. 3). The 16S rDNA sequence data indicated that the suspended and biofilm community comprised < 10% methanogens. The hydrogenotrophic methanogens were more abundant than the acetoclastic methanogens in the suspended biomass 26 days after start-up and in the biofilm biomass at the end of Phase 2 (Fig. S5A), suggesting the hydrogen utilization pathway became more important after biomass adaptation to the psychrophilic temperature. A shift towards hydrogenotrophic methanogenesis has also been observed previously in other anaerobic systems when transitioning from mesophilic to psychrophilic conditions using DNA-based analyses and specific methanogenic activity assays (McHugh *et al*., 2013; Collins *et al*., 2006; Connaughton *et al*., 2006), and has been explained by increased hydrogen solubility and thus increased substrate availability for hydrogenotrophic metabolisms (Lettinga *et al*., 2001). In contrast to 16S rDNA sequence data, 16S rRNA sequencing indicated that the relative activity of acetoclastic and hydrogenotrophic methanogens was similar in our system (Fig. S5B). MF and HF biofilms also had substantially greater activity of methanogens relative to the suspended biomass and LF biofilm on day 138. Specifically, methanogens represented 33% and 34% of the combined bacterial and archaeal activity in MF and HF biofilms, respectively, in comparison to only 15% in the suspended biomass and LF biofilm (Fig. 3). These observations correlated with the low levels of acetate and propionate and high dissolved methane oversaturation by the end of Phase 2 (Fig. 2), and suggest that a high level of methanogenesis occurred in the MF and HF biofilms.

*Methanosaeta* is believed to be the only methanogen that exclusively produces methane through the acetoclastic pathway (Smith and Ingram-Smith, 2007). In the suspended biomass, the relative abundance of *Methanosaeta* spp. decreased from 1.6% on day 0 to 0.55% on day 138 (Fig. 3A). However, their relative activity was fairly stable (Fig. 3B), resulting in an increase in the activity/abundance ratio over time. The consistently low relative abundance and high relative activity of *Methanosaeta* spp. in the suspended biomass suggest that growth was negligible, possibly due to the psychrophilic temperature. Further, the observation of long-term (over 138 days) stable suspended biomass concentrations (Fig. S4) supports this notion.

*Methanosarcina* produces methane from acetate, hydrogen and C1 compounds (Mladenovska and Ahring, 1997; Welander and Metcalf, 2005) and has thus been categorized as mixotrophic. The relative activity of *Methanosarcina* spp. increased over time in the suspended biomass and comprised 18% and 21% of relative methanogenic activity in MF and HF biofilm biomass respectively (Fig. S5). *Methanosarcina* spp. were either not detected or detected at ≤ 0.23% of the combined bacterial and archaeal abundance (Fig. 3A). Similarly, *Methanosarcina* spp. were not detected via 16S rDNA sequencing in another psychrophilic AnMBR study (Bandara *et al*., 2012) and were detected at < 0.50% of the archaeal community in our previous AnMBR work at 15°C (Smith *et al*., 2013). *Methanosarcina* has a greater maximum growth rate and half-saturation coefficient than *Methanosaeta*, which often leads to the dominance of *Methanosaeta* when acetate concentrations are low, such as in continuously fed anaerobic digestion (Conklin *et al*., 2006) or during low-strength wastewater treatment. It is unclear why the activity of *Methanosarcina* was high in this study, particularly in the MF and HF biofilms, as the acetate concentration was below or within the reported range of threshold levels at which *Methanosarcina* spp. are typically inactive (11–71 mg l^−1^ acetate; Jetten *et al*., 1992) (Fig. 2B). Psychrotolerant *Methanosarcina* spp. have been observed in the environment (Simankova *et al*., 2001; von Klein *et al*., 2002), but a specific mechanism for low-temperature adaptation that would give a competitive advantage over *Methanosaeta* or other methanogens in psychrophilic AnMBR has not been reported. However, *Methanosarcina* spp. have a unique surface structure (Francoleon *et al*., 2009; De Vrieze *et al*., 2012), which may aid in cell attachment to surfaces (Robinson *et al*., 2012; De Vrieze *et al*., 2012). We hypothesize that the metabolic flexibility and unique surface structure of *Methanosarcina* spp. offered a competitive advantage in the biofilm relative to other methanogens.

*Methanoregula* spp. and *Methanospirillum* spp. were the dominant active hydrogenotrophic methanogens classified in suspended and biofilm biomass and comprised 24% and 14% of methanogenic activity in the HF biofilm biomass respectively (Fig. 3B). *Methanoregula* spp., mesophilic hydrogenotrophic methanogens, were only recently cultivated from a full-scale upflow anaerobic sludge blanket reactor (Yashiro *et al*., 2011) and an acidic peat bog (Bräuer *et al*., 2011). Growth for both of these *Methanoregula* spp. was demonstrated at temperatures as low as 10°C, suggesting tolerance to psychrophilic temperature. The activity/abundance ratio of *Methanoregula* spp. was 0.41 and 0.33 in MF and HF biofilm biomass, respectively, whereas the ratio for *Methanospirillum* spp. was 2.5 and 2.0 in MF and HF biofilm biomass, respectively, suggesting that the activity per cell for *Methanospirillum* spp. was significantly greater than for *Methanoregula* spp. in the biofilm biomass.

It is important to note the limitations of our approach to infer microbial abundance and activity. 16S rRNA operon number varies from 1 to 15 copies per genome (Klappenbach *et al*., 2000), which can lead to over or under-representation of specific phylogenies if a constant operon number is assumed across all phylogenies (Větrovský and Baldrian, 2013). Normalization of sequencing results to operon number is challenging since the operon number is not available for all methanogens (Lee *et al*., 2014), and variations in operon number exist at the phylogenetic resolution provided by our sequencing data (species level). Variations in 16S rRNA abundance between phylogenies based on cell size and other factors are another concern with our approach. 16S rRNA abundance is also not directly linked to a specific cellular function (e.g. methanogenesis) and does not always correlate well with activity even for pure cultures under steady-state conditions (Blazewicz *et al*., 2013). Further, inactive or dormant microorganisms sometimes contain high amounts of rRNA (Sukenik *et al*., 2012). Another limitation is the lack of absolute abundance or gene expression data, which can only be obtained when accurate quantitative nucleic acid extraction is possible. Quantitative DNA and RNA extractions are particularly challenging when working with biomass from environmental systems with different characteristics, such as suspended and biofilm biomass in this study. For example, elevated concentrations of extracellular polymeric substances in biofilm biomass (Smith *et al*., 2013) may reduce extraction efficiency and quality of nucleic acids extracted. Such matrix effects may influence microbial characterization due to biases in nucleic acids extraction, PCR, reverse transcription or other steps (Martin-Laurent *et al*., 2001). These concerns were motivation for validating our approach by monitoring changes in the expression of the *mcrA* gene in methanogens (Thauer, 1998).

**Figure 4:**
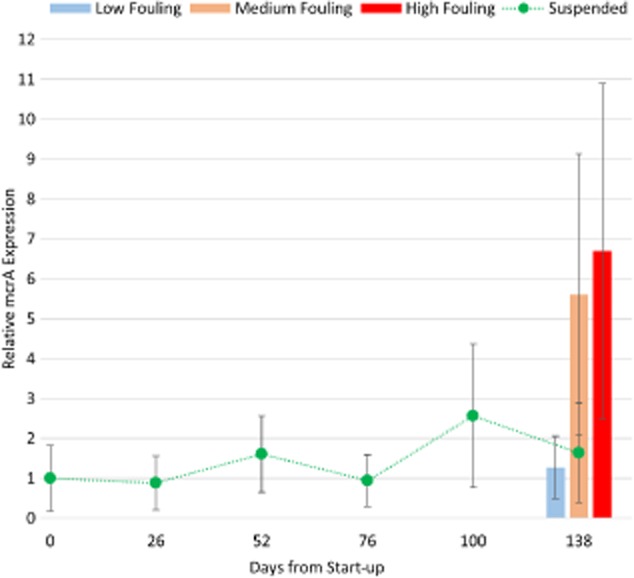
Relative expression of *mcrA* in suspended and biofilm biomass. Copies of *mcrA* transcripts in each biomass sample were first normalized to 16S rRNA copies. Next, relative *mcrA* expression was calculated by normalizing the ratio of *mcrA* transcript copies to 16S rRNA copies in each sample to the corresponding ratio in the suspended biomass sample collected on day 0. Error bars represent the standard deviation of the ratio of triplicate qPCR reactions at serial dilutions of cDNA template concentration.

The RT-qPCR results quantifying *mcrA* transcripts correlated well with performance observations and 16S rRNA sequence data, indicating significantly higher methanogenic activity in MF and HF biofilm biomass relative to suspended or LF biofilm biomass (Fig. 4). Taken together, our results provide strong evidence that the membrane biofilm was enriched in highly active acetoclastic and hydrogenotrophic methanogens.

### Phylogenetically distinct syntrophic bacterial operational taxonomic unit (OTU) was highly active in the biofilm

**Figure 5:**
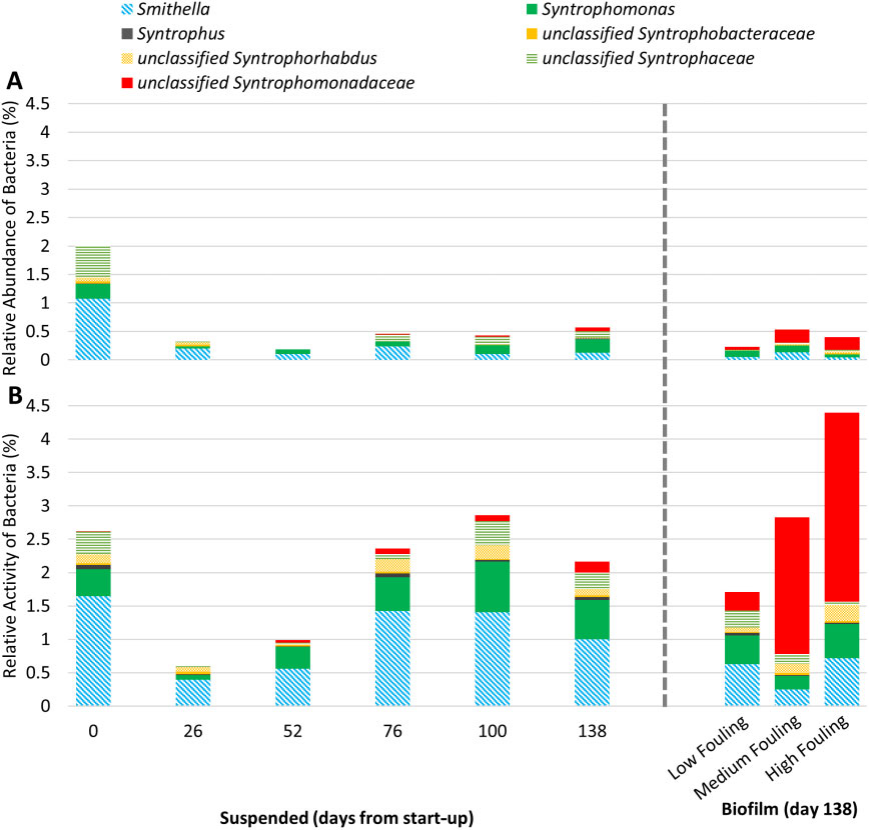
(A) Relative abundance of known fatty acid-oxidizing obligate syntrophic bacteria identified at the genus level or family level based on 16S rDNA sequencing and (B) relative activity of syntrophic bacteria identified at the genus level or family level based on 16S rRNA sequencing. Data were obtained for suspended biomass samples collected from start-up to the end of Phase 2 and in biofilm samples obtained at the end of Phase 2. Data are expressed as percentages and were normalized using the total number of 16S rDNA bacterial sequences (A) and 16S rRNA bacterial sequences (B). A truncated y-axis (0–4.5%) is shown to accentuate changes in abundance and activity.

The relative activity of fatty acid-oxidizing obligate syntrophic bacteria correlated well with the differing VFA concentrations in the permeates (Fig. 5). On day 0, a high level of syntrophic activity was observed in the suspended biomass, which quickly fell to 0.33% activity of total bacteria by day 26, likely due to the introduction of a mesophilic inoculum into a system with a psychrophilic operational temperature. In addition, the calculated average velocity gradient (g) due to biogas sparging in our system of 410 s^−1^ is much higher than recommended g values for effective mixing in anaerobic digestion (50–80 s^−1^; Tchobanoglous *et al*., 2003), and raises the question of how this high shear affected the microbial community and syntrophic associations in particular. Research has suggested that high shear can be detrimental to anaerobic digester performance under high loading rates due to increased hydrolysis and fermentation resulting in acidification (Stroot *et al*., 2001; Vavilin and Angelidaki, 2005; Padmasiri *et al*., 2007). Hoffmann and colleagues (2008) demonstrated that even when loading rates were low, high shear conditions had a detrimental effect on digester performance. None of these studies conclusively determined how high shear conditions may impact anaerobic microbial communities, and the effects of shear in AnMBRs operated at low loading rates, such as the system in this study, have not been studied. The relative activity levels of syntrophic bacteria in the suspended biomass gradually increased over time and showed differences in the three biofilm samples collected on day 138, consistent with AnMBR performance observations and methanogenic activity (Fig. 5). Known syntrophic acetate-oxidizing bacteria (Hattori, 2008) were not detected in the biofilm, suggesting that acetate removal was primarily via acetoclastic methanogenesis.

An OTU unclassified at the genus level according to the Ribosomal Database Project (RDP) belonging to the family *Syntrophomonadaceae* comprised a significant proportion of the relative activity of all identified syntrophs in MF and HF biofilm biomass (i.e. 72% and 64% respectively), but accounted for only 7.7% of the relative activity of syntrophs in the suspended biomass on day 138. A representative sequence from this OTU exhibited 95% identity with *Syntrophomonas zehnderi*, an obligate syntrophic microorganism (Sousa *et al*., 2007). Interestingly, genera in *Syntrophomonadaceae* have only been observed to syntrophically oxidize C4 compounds (e.g. butyrate) and higher order organics (Stams *et al*., 2012). In our system, butyrate concentrations were below the detection limit, and thus it is surprising that a butyrate oxidizing syntroph would have such high activity in the biofilm particularly relative to propionate oxidizing syntrophs (e.g. *Smithella*) given the significant propionate removal in the biofilm. The unclassified OTU may be a yet to be described species of *Syntrophomonadaceae* capable of C3 oxidation. Alternatively, Gan and colleagues (2012) using DNA-based stable isotope probing proposed a novel pathway in which *Smithella* spp. first dismutate propionate to acetate and butyrate followed by butyrate oxidation by *Syntrophomonas* spp. via a trophic interaction. Based on the significant removal of propionate in the biofilm, non-detectable levels of butyrate in the bioreactor, and activity of both *Smithella* spp. and the unclassified OTU belonging to *Syntrophomonadaceae*, it is possible that this novel pathway occurred here. In this scenario, butyrate may have remained non-detectable acting as a transient metabolite. This scenario would require cooperation between two syntrophic bacteria and a hydrogenotrophic methanogen, and thus may benefit from the increased spatial organization afforded to the biofilm community relative to the suspended biomass. The unclassified OTU may also be more active in the biofilm than in the suspended biomass due to differential preferences in growth mode (i.e. attached versus suspended).

The AnMBR biofilm may support other syntrophic interactions. The sulfate reducer *Desulfovibrio vulgaris* has been identified as capable of growing syntrophically with a hydrogenotrophic methanogen on lactate in the absence of sulfate (Scholten *et al*., 2007). The majority of sulfate reduction in our system occurred in the suspended biomass with sulfate concentrations < 1 mg l^−1^ in the bioreactor at the end of Phase 2 (prior to biofilm biomass sampling). The relative activity of some sulfate reducers correlated well with performance data. For example, *Desulfobulbus* spp. had a relative activity of 2.2% in the suspended biomass but only 0.25% in HF biofilm biomass (Fig. S6). However, the relative activity of *Desulfovibrio* spp. was greater in the biofilm biomass, 7.2% versus 4.3% in HF biofilm and suspended biomass, respectively, despite limited sulfate reduction across the biofilm. It is possible that, in the absence of sulfate, *Desulfovibrio* spp. transition to syntrophic metabolisms potentially enhanced in the biofilm. The biofilm may also provide an environment conducive to DIET (Summers *et al*., 2010; Morita *et al*., 2011). However, *Geobacter* spp., a genus with members likely to participate in DIET (Summers *et al*., 2010), had lower relative activity in the biofilm than in the suspended biomass. The highly active unclassified *Syntrophomonadaceae* OTU could potentially be involved in DIET, although genome sequencing of *Syntrophomonas wolfei* did not identify the outer membrane cytochromes necessary for DIET (Sieber *et al*., 2010). The potential role of DIET in AnMBR domestic wastewater treatment has yet to be explored and deserves further investigation.

We hypothesize that reduced mass-transfer limitations, increased substrate availability and spatial organization of the biofilm community may all play a role in the high microbial activity observed in the biofilm. It is important to note that microbial activity in the biofilm could also be influenced by elevated temperatures due to exothermic reactions occurring there. To estimate the maximum potential temperature differential inside the biofilm relative to the suspended biomass, we considered hydrogenotrophic methanogenesis, an exothermic reaction, in accordance with the measured permeate dissolved methane oversaturation and reaction enthalpies as discussed by Westermann (1994). The calculated maximum heat differential was < 0.2°C, suggesting a negligible difference between biofilm and suspended biomass temperature. Further, other reactions occurring in the biofilm (i.e. acetoclastic methanogenesis and propionate oxidation) are endothermic. Future research should evaluate the underlying mechanism(s) responsible for the high microbial activity in the biofilm.

### Biofilm treatment performance is maintained in the absence of TMP

During Phases 3 and 4, biogas sparging on HF was increased to evaluate if biological treatment in the biofilm could be maintained without high TMP (i.e. HF operation was switched to LF operation, and is indicated in Fig. 1 as HF–LF). The HF–LF permeate COD during Phases 3 and 4 averaged 24 ± 7.1 mg l^−1^ (Fig. S7), a similar effluent quality to that obtained at the end of Phase 2. Further, HF–LF permeate propionate concentration was below detection by the start of Phase 4 (Fig. S8), implying that the activity of syntrophic propionate oxidizers improved during this time period, despite low TMP. Dissolved methane oversaturation remained high, averaging 2.2 ± 0.49, and was thus primarily driven by biological activity in the biofilm rather than high TMP or a combination of the two. One concern with our comparative evaluation is that pump slippage from high TMP resulted in reduced flux for HF, which increased substrate contact time in the biofilm and could have affected our observations. However, HF flux was restored after returning TMP to near zero, suggesting that the potentially higher substrate contact time did not impact our comparison. These results demonstrate that biofilm activity can be maintained in the absence of TMP and suggest that the active microbial community in the biofilm is tightly adhered to the membrane surface. The active community is either distinct from the layer of foulants contributing to high TMP or has sufficient biological activity to maintain treatment under LF conditions (i.e. less biomass).

After restoring the TMP in the MF membrane unit to near zero during Phase 3 (indicated as MF–LF in Fig. 1), fouling for this membrane was increased during Phase 4 in an attempt to replicate the performance obtained previously with HF (indicated as MF–HF in Fig. 1). The MF–HF permeate COD during Phase 4 was 37 ± 7.0 mg l^−1^, approaching an effluent quality similar to that of the HF permeate in Phase 2. Dissolved methane oversaturation in the MF–HF permeate increased, averaging 2.6 ± 0.68. These observations provide evidence that biofilm promotion via reduced biogas sparging to enhance treatment performance is replicable.

Because we were able to maintain biological activity after returning to near zero TMP, biofilm promotion strategies may only require an inoculation period in which the membrane is colonized from the suspended biomass and can then be operated at low TMP. Long-term operation with substantial membrane fouling is undesirable from an operation’s standpoint, and thus our demonstration of biofilm activity at low TMP is encouraging. The industry’s current reliance on aggressive chemical cleaning in membrane installations disrupts the active biofilm community and may have prevented similar observations in the past. Since we were able to return to a low TMP after extended periods of fouling by solely adjusting biogas sparging flow rate without chemical cleaning, this fouling control method or an alternative strategy could be explored in full-scale systems to sustain biological activity in the biofilm. The energy demands of a higher biogas sparging flow rate to do so need to be weighed against the benefits of operation without chemical cleaning.

### Biofilm development is an attractive operational strategy for low-temperature AnMBR

We have shown that effluent quality in AnMBR domestic wastewater treatment can be improved by rethinking common perceptions of membrane fouling. Multiple lines of evidence (i.e. 16S rRNA sequencing, RT-qPCR targeting the *mcrA* gene and performance observations) show that controlled membrane fouling leads to the development of a membrane biofilm enriched in highly active acetoclastic and hydrogenotrophic methanogens and syntrophic bacteria. This active biofilm may also have additional unexplored benefits in AnMBR, such as removal of antibiotic resistance genes (e.g. as demonstrated in aerobic membrane bioreactors; Riquelme Breazeal *et al*., 2012) or other micropollutants. Future research should evaluate the underlying mechanisms behind the biofilm community’s high biological activity (e.g. reduced mass transfer limitations, lower intercellular distances or other factors), the impact of biofilm promotion strategies on long-term membrane fouling and the biofilm’s response to chemical membrane cleaning. It is important to note that this research was performed using synthetic domestic wastewater and that evaluation using real domestic wastewater is necessary. Future research is also required to develop low-energy dissolved methane recovery technologies to prevent greenhouse gas emissions, particularly when biofilm activity results in significant oversaturation.

## Experimental procedures

### AnMBR operation and chemical assays

A bench-scale AnMBR described previously (Smith *et al*., 2013) was redesigned to incorporate three submerged flat-sheet membrane housings with microfiltration polyethersulfone membranes (GE Osmonics, Greenville, SC) at a pore size of 0.2 μm and a total effective membrane area of 924 cm^2^. The system was operated at 15°C with a synthetic domestic wastewater (Smith *et al*., 2013). Reactor temperature was controlled using a water jacket connected to a Polystat 6-L re-circulating water bath (Cole-Parmer, Vernon Hills, IL). Water bath temperature was adjusted based on temperature measurement of a submerged probe located in close proximity to the membrane surface. Three mini diaphragm pumps (KNF Neuberger, Trenton, NJ) re-circulated headspace biogas and dispersed it directly below each membrane housing by horizontally placed sparging tubes for fouling control. Biogas sparging flow rates were independently controlled for each membrane housing using in-line flow metres. The liquid volume of the reactor was 4 l. The AnMBR was inoculated with sludge from a mesophilic (32°C) wastewater treatment plant anaerobic sludge digester (Northfield Wastewater Treatment Plant, Whitmore Lake, MI) at an initial volatile suspended solid concentration of approximately 8000 mg l^−1^.

The system was operated at a target hydraulic retention time (HRT) of 16 h, which corresponded to an organic loading rate of 670 mg COD/l●d. Biomass was only removed from the AnMBR for sampling purposes, which resulted in an SRT of approximately 300 days. From days 1 through 99 (Phase 1), a membrane flux of 2.7 l m^−2^ h^−1^ (LMH) was targeted for each membrane housing. This relatively low membrane flux ensured operation with minimal membrane fouling could be maintained without chemical cleaning and provided good operational control. The high biogas sparging flow rate selected for Phase 1 (3.0 l biogas min^−1^ for each membrane housing or 5.8 m^3^ biogas h^−1^ m^−2^ membrane surface area) helped prevent the formation of a membrane biofilm. Backflushing was performed for 30 s every 10 min of bioreactor operation. Due to pump slippage at high TMP (Phases 2 and 4), the flux for P1 was increased as necessary to maintain an HRT of 16 h. Data from days 139 to 151 are not presented due to a brief exposure of the system to air during biofilm sampling on day 138 (described below), which resulted in poor system performance. Chemical assays were performed as described in Appendix S1.

### Biomass samples and nucleic acids extraction

Suspended biomass samples from the AnMBR were taken on days 0, 26, 52, 76, 100 and 138 of operation, pelletized by centrifugation at 5000× *g* for 5 min at 4°C, and immediately stored at −80°C after decanting the supernatant. Biofilm biomass samples were gently scraped from the membrane surface of P1 (LF), P2 (MF) and P3 (HF) on day 138 using sterile lazy-l spreaders, pelletized, decanted and immediately stored at −80°C. P1 (LF) had limited biofilm biomass, which was loosely associated with the membrane, consistent with the LF condition. Biomass samples for RNA extraction were prepared similarly except for the addition of RNAlater (Qiagen, Valencia, CA) prior to storage. DNA and RNA extractions were accomplished as described in Appendix S2.

### RT-qPCR

Primers targeting the *mcrA* gene were designed via an *in silico* analysis described in Appendix S3. Universal primers targeting the V4 region of the 16S rDNA (Caporaso *et al*., 2011) were used to quantify 16S rRNA for normalization of *mcrA* transcript quantification. Coverage of 16S rRNA primers was verified using TestPrime 1.0 (Klindworth *et al*., 2012) (Tables S1 and S2).

Reverse transcription to generate single-stranded complementary DNA (cDNA) from RNA extracts was performed using the SuperScript VILO cDNA Synthesis Kit according to manufacturer’s instruction (Life Technologies, Grand Island, NY). Two-step RT-qPCR, as opposed to one-step in which cDNA synthesis and qPCR occur sequentially in one reaction, was done to allow for sequencing of synthesized cDNA (described below).

Standards for RT-qPCR were prepared as described in Appendix S4. Reverse transcription-qPCR was conducted on a Mastercycler realplex ep (Eppendorf, Hamburg, Germany) with a total reaction volume of 20 μl as described in Appendix S4. The R^2^ and efficiencies for *mcrA* and 16S rRNA standard curves were 0.991 and 0.997 and 75% and 71% respectively.

### 16S rDNA and rRNA sequencing

Polymerase chain reaction, sample multiplexing and Illumina MiSeq sequencing of 16S rDNA and rRNA were performed by the Host Microbiome Initiative (University of Michigan, Ann Arbor, MI; further information provided in Appendix S5). After quality filtering and subsampling, 16 587 paired-end reads (2 × 250 bp) per sample were generated. The resulting sequences were processed with mothur (Schloss *et al*., 2009) following the Schloss MiSeq SOP and classified using the Ribosomal Database Project (Maidak *et al*., 2013) and Basic Local Alignment Search Tool (blast; NCBI, Bethesda, MD). These sequence data have been submitted to the DDBJ/EMBL/GenBank databases under Accession Number SRP056737.

## Supporting Information

**Fig. S1.** Influent (total and soluble), bioreactor (soluble) and permeate COD during days 1–100. **Fig. S2.** P1 VFA concentrations (concentrations are expressed as the actual compound, not as COD), theoretical COD contribution from measured VFAs and measured COD during days 1–100. Total as COD is the calculated theoretical COD contribution from measured VFAs. Results for P2 and P3 were very similar (data not reported). Error bars represent standard deviations of triplicate IC injections. **Fig. S3.** COD mass balance for days 100–138. Total COD_out_ is the summation of measured permeate COD, measured dissolved methane, measured gaseous methane, theoretical COD removal from measured sulfate reduction and theoretical COD from measured biomass wasting. **Fig. S4.** Total suspended solids (TSS) and volatile suspended solids (VSS) in the bioreactor during days 1–173 (primary y-axis) and inverse Simpson index in suspended biomass based on 16S rDNA sequencing (secondary y-axis). Error bars represent standard deviation of triplicate sample analysis. **Fig. S5.** (A) Relative abundance of methanogens identified at the genus level based on 16S rDNA sequencing and (B) relative activity of methanogens identified at the genus level based on 16S rRNA sequencing in suspended biomass from start-up to the end of Phase 2 and in biofilms at the end of Phase 2. Data are expressed as a percentage and were normalized using the total archaeal 16S rDNA sequences (A) and 16S rRNA sequences (B). **Fig. S6.** (A) Relative abundance of sulfate-reducing bacteria identified at the genus level based on 16S rDNA sequencing and (B) relative activity of sulfate-reducing bacteria identified at the genus level based on 16S rRNA sequencing in suspended biomass from start-up to the end of Phase 2 and in biofilms at the end of Phase 2. Data are expressed as a percentage and were normalized using the total 16S rDNA sequences (A) and 16S rRNA sequences (B) (including archaeal and bacterial sequences). A truncated y-axis (0–8%) is shown to accentuate changes in abundance and activity. **Fig. S7.** Influent (total and soluble), bioreactor (soluble) and permeate COD during Phases 3 and 4. **Fig. S8.** P1, P2 and P3 permeate VFA concentrations (concentrations are expressed as the actual compound, not as COD), theoretical COD contribution from measured VFAs, and measured COD during Phases 3 and 4. Total as COD is the calculated theoretical COD contribution from measured VFAs. Error bars represent standard deviations of triplicate IC injections. **Table S1.** Primer coverage of *Archaea* for 16S rDNA primers F515 (GTGCCAGCMGCCGCGGTAA) and R806 (GGACTACHVGGGTWTCTAAT) targeting the V4 region (Caporaso *et al*., 2011) according to TestPrime 1.0. TestPrime 1.0 evaluates the coverage of primer pairs by running an *in silico* PCR using the SILVA databases. Zero primer mismatches were allowed. **Table S2.** Primer coverage of *Bacteria* for 16S rDNA primers targeting the V4 region according to TestPrime 1.0 (see Table S1 legend for additional details). The coverage of taxa with known fatty acid-oxidizing syntrophic bacteria is specified down to the genus or family levels. **Appendix S1.** Chemical assays for process monitoring. **Appendix S2.** DNA and RNA extractions. **Appendix S3.** Primer design for *mcrA* gene. **Appendix S4.** RT-qPCR. **Appendix S5.** Illumina sequencing. **Appendix S6.** Start-up of psychrophilic AnMBR.
