# Supplementary material for: Membrane biofilm development improves COD removal in anaerobic membrane bioreactor wastewater treatment
Source: Microb Biotechnol. 2015 Aug 4;8(5):883–94. doi: 10.1111/1751-7915.12311 (PMC4554476; doi:10.1111/1751-7915.12311)
Supplement: Supplementary file 1 [file mbt20008-0883-sd1.docx]

***Supporting Information***

**Supporting Text 1 – Chemical Assays for Process Monitoring**

COD, total suspended solids (TSS), and VSS were determined using procedures outlined in Standard Methods (APHA 2005). Soluble COD was determined after filtering samples through a 0.2 µm filter to be consistent with the physical removal of suspended material in the AnMBR by membrane filtration (same pore size). Concentrations of volatile fatty acids (VFAs) (formate, acetate, propionate, butyrate, and valerate) and sulfate were determined with an ion chromatograph (ICS-1600, Dionex, Sunnyvale, CA) equipped with a conductivity detector, autosampler, and reagent free eluent generator to produce a KOH gradient. Eluent was passed through a Dionex AS-11HC column at 60°C at a flow rate of 0.30 mL/min.

Biogas methane content was measured with a gas chromatograph (Gow-Mac, Bethlehem, PA) coupled with a thermal conductivity detector (TCD). Measurement of dissolved methane in the permeate was accomplished as previously described (Rudd et al 1974). Briefly, 30 mL of permeate were collected in a syringe containing 30 mL nitrogen gas. The syringe was shaken by hand for 1 minute to strip dissolved methane into the gas phase. The gas phase was collected from the syringe and sampled for gas chromatography. Biogas production was measured by collecting gas in a 1-L Tedlar bag and quantifying the production daily using a wet-type gas meter (Actaris Metering Systems, Dordrecht, The Netherlands).

Permeate sampling was performed approximately daily for measurements of COD, VFAs, and sulfate, and every two to four days for dissolved methane content. Influent and bioreactor content were collected every two to four days to measure COD and VFAs, and weekly for TSS and VSS. Biogas content was determined every two to four days. Samples for soluble COD, VFA, and sulfate analyses were immediately filtered through a 0.2 μm-filter after sampling and preserved using concentrated sulfuric acid (COD) or 1 M sodium hydroxide (VFAs and sulfate) and stored at 4°C for up to 10 days.

**Supporting Text 2 – DNA and RNA Extractions**

DNA extraction from pelletized biomass was performed by three 2-min bead beating steps (Mini-Beadbeater-96, BioSpec Products, Bartlesville, OK) with 0.1 mm diameter silicon beads in lysis buffer, proteinase K digestion, and automated extraction using the Maxwell 16 Blood LEV kit according to manufacturer’s instruction (Promega, Madison, WI). RNA extraction was performed by three 2-min bead beating steps with 0.1 mm diameter silicon beads in 1-thiolyglycerol homogenization buffer and automated extraction using the Maxwell 16 simplyRNA tissue kit according to manufacturer’s instructions except that 10 µL of DNase 1 (instead of 5 µL) was used. DNA and RNA quality and quantity were assessed via spectrophotometry (Nanodrop 1000, Thermo Fisher Scientific, Wilmington, DE) and the Quantifluor dsDNA and RNA systems (Promega, Madison, WI), respectively, with a fluorospectrometer (Nanodrop 3000, Thermo Fisher Scientific, Wilmington, DE). Select RNA extracts were further analyzed for quality via automated electrophoresis using the Experion RNA analysis kit (Bio-Rad, Hercules, CA).

**Supporting Text 3 – Primer Design for *mcrA* Gene**

An *in silico* analysis of primers targeting the *mcrA* gene (Juottonen et al 2006, Steinberg and Regan 2008, Steinberg and Regan 2009, Zeleke et al 2013) was performed in MEGA (Tamura et al 2013) using partial *mcrA* gene sequences available in GenBank (NCBI, Bethesda, MD) and back-translated full-length *mcrA* protein sequences generated by EMBOSS Backtranseq with the *Methanothermobacter thermautotrophicus* strain Delta H codon usage table (EMBL-EBI, Hinxton, UK). Based on the *in silico* analysis, additional primer degeneracies were incorporated into forward primer mlas (Steinberg and Regan 2009) to provide greater coverage (5’-GGYGGTGTMGGNTTCACHCARTA-3’). Primer specificity was assessed using MFE-primer 2.0 (Qu et al 2012). Reverse primer mcra-rev was used as reported in the literature (5’-CGTTCATBGCGTAGTTVGGRTAGT-3’) (Steinberg and Regan 2008). A detailed discussion of primer design and characterization is provided elsewhere (Clancy et al 2014).

**Supporting Text 4 – RT-qPCR**

RT-qPCR standards were prepared by amplifying *mcrA* and 16S rRNA genes and cDNA synthesized transcripts from a pool of 21 DNA and 21 cDNA samples from the bench-scale AnMBR pooled by equal mass (He and McMahon 2011, Sonthiphand et al 2013). PCR to prepare RT-qPCR standards was performed in 20 µL reactions using the above described primer sets at 500 nM, 0.5 ng of pooled template, 0.3 mg/mL bovine serum albumin (BSA), 10 µL Phusion High-Fidelity Master Mix (NEB, Ipswich, MA), and ultra-pure nuclease free water. Thermocycling conditions to prepare RT-qPCR standards consisted of an initial 2 min denaturation at 95°C, followed by 30 cycles of denaturing at 95°C for 20 s, annealing at 55°C for 15 s, and extension at 72°C for 30 s, followed by a final extension at 72°C for 5 min. PCR products were run on a 1.5% agarose gel, and bands were excised with a sterile scalpel and purified using the QIAquick Gel Extraction Kit (Qiagen, Valencia, CA). The purified amplicon pool was quantified via Quantifluor dsDNA system and fluorospectrometry. Serial dilutions of the purified amplicon pools were prepared as RT-qPCR standards from 10^8^ to 10^3^ copies.

Samples were quantified in triplicate using serial dilutions of template. All standards and a no template control were run in triplicate. Each reaction contained 2 µL template, 500 nM forward and reverse primers, 10 µL 2X Fast-Plus EvaGreen Master Mix (Biotium, Hayward, CA), and ultra-pure nuclease free water. Thermocycling conditions for RT-qPCR targeting 16S rRNA of samples were the same as described above for standards preparation, except that 50 cycles were performed instead of 30. For *mcrA* quantification in samples, thermocycling conditions consisted of an initial 2 min denaturation at 95°C, followed by 5 cycles of denaturing at 95°C for 20 s, annealing at 55°C for 15 s, a temperature ramp at a rate of 0.1°C/s to 72°C to aid in annealing due to the highly degenerate nature of the primer set (Luton et al 2002, Morris et al 2014), and extension at 72°C for 30 s, followed by 45 cycles without the temperature ramp and lastly, a final extension at 72°C for 5 min. In addition, a melt curve analysis was performed to verify qPCR specificity.

**Supporting Text 5 – Illumina Sequencing**

PCR and sequencing of 16S rDNA and rRNA was accomplished using a universal 16S rDNA primer set targeting the V4 region (Caporaso et al 2011) barcoded and using sequencing primers described in Kozich et al. (2013). PCR reactions were 20 µL and included primers at 500 nM, 10 µL 2x Accuprime buffer 11 (Invitrogen, Carlsbad, CA), 0.15 µL Accuprime Hi Fi TAQ, 0.5 ng template, and nuclease-free water. Thermocycling conditions consisted of an initial 2 min denaturation at 95°C, followed by 30 cycles of denaturing at 95°C for 20 s, annealing at 55°C for 15 s, and extension at 72°C for 5 min, followed by a final extension at 72°C for 5 min. Amplicons were pooled by equal mass using the SequalPrep Normalization Plate Kit (Life Technologies, Grand Island, NY). Multiplexed amplicons were sequenced via Illumina MiSeq using the MiSeq Reagent Kit V2 (2x250 bp reads) and sequencing primers described in Kozich et al. (2013).

Table S1. Primer coverage of *Archaea* for 16S rDNA primers F515 (GTGCCAGCMGCCGCGGTAA) and R806 (GGACTACHVGGGTWTCTAAT) targeting the V4 region (Caporaso et al. 2011) according to TestPrime 1.0. TestPrime 1.0 evaluates the coverage of primer pairs by running an *in silico* PCR using the SILVA databases. Zero primer mismatches were allowed.


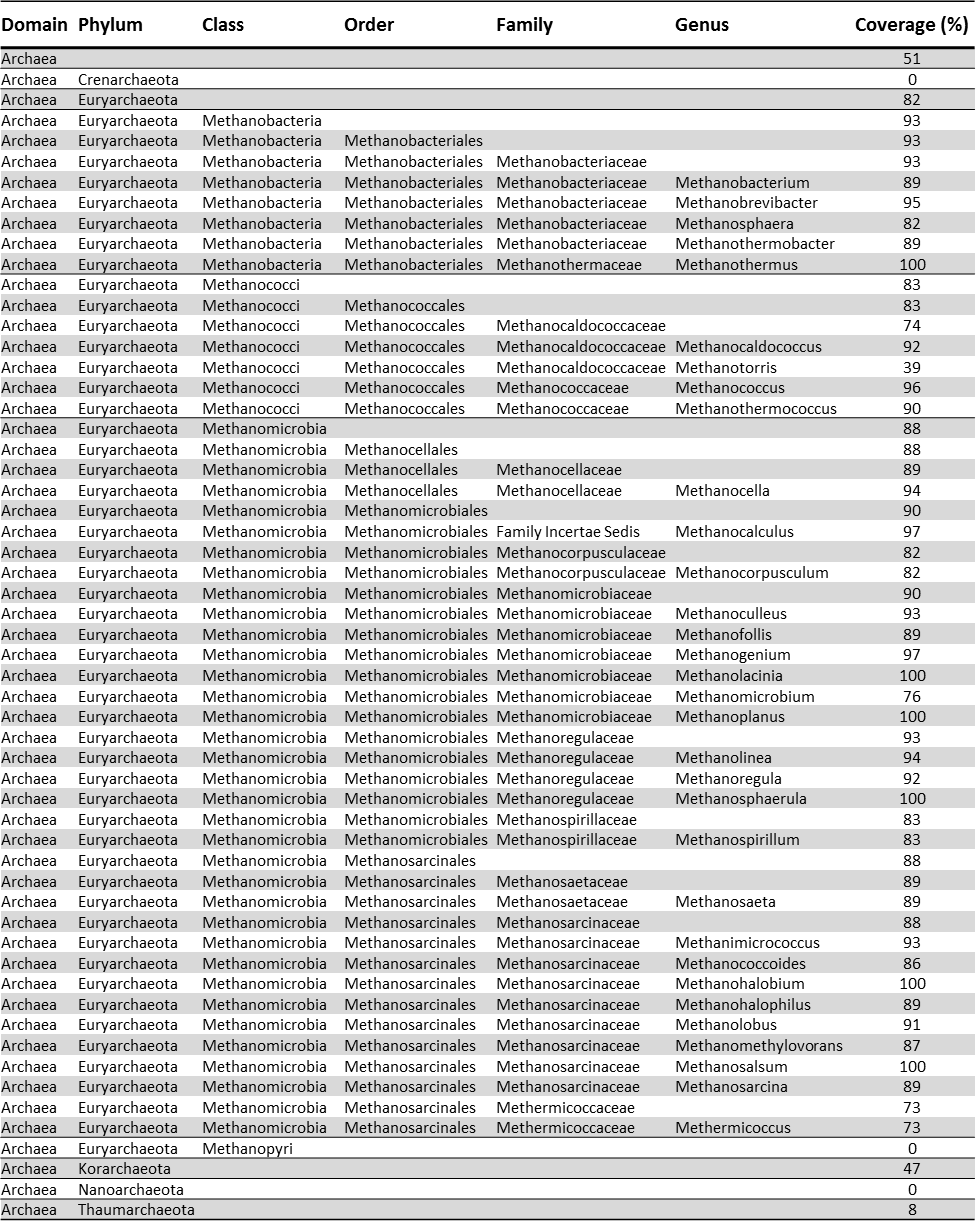


Table S2. Primer coverage of *Bacteria* for 16S rDNA primers targeting the V4 region according to TestPrime 1.0 (see Table S1 legend for additional details). The coverage of taxa in which known fatty acid-oxidizing syntrophic bacteria group is specified down to the genus or family levels.


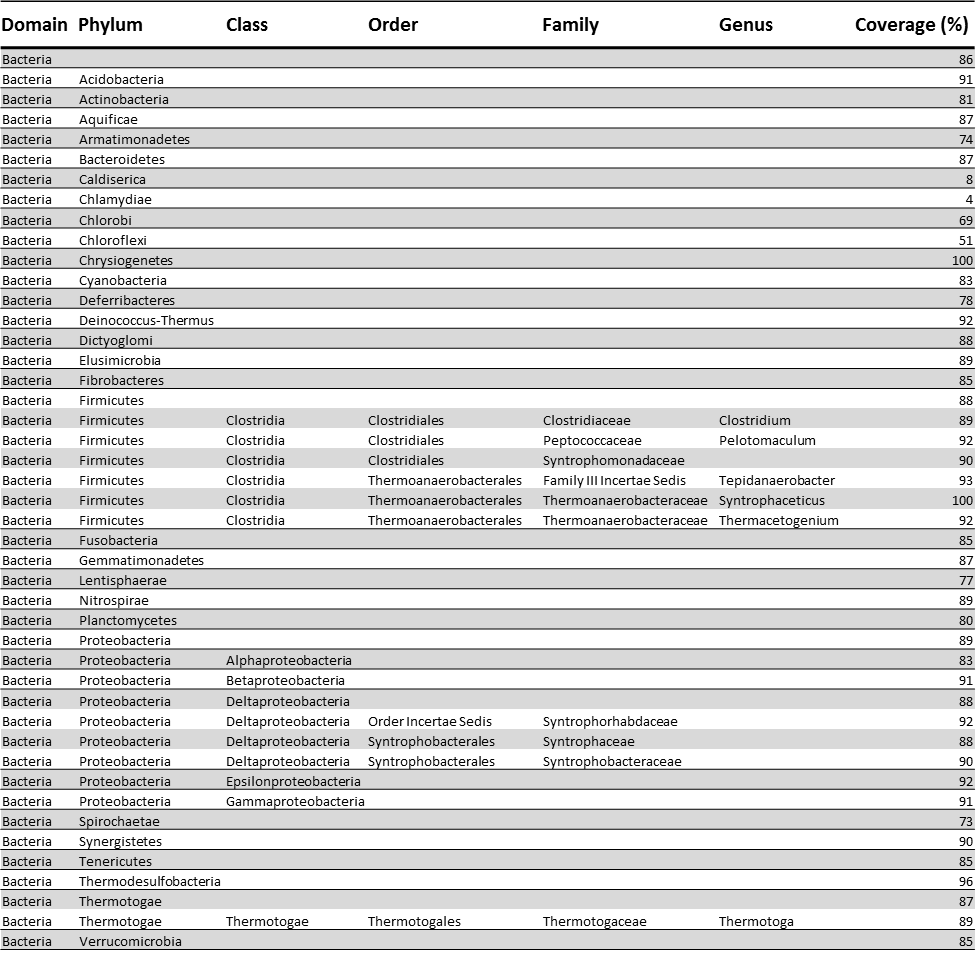


**Supporting Text 6 - Startup of psychrophilic AnMBR**

In a previous study, we inoculated a psychrophilic AnMBR with a mixture of two mesophilic sources and one psychrophilic source (Smith et al 2013). Pyrosequencing of 16S rDNA in each inoculum and AnMBR biomass samples collected after one year of operation suggested that the AnMBR microbial community structures were most similar to the mesophilic inocula. Therefore, we elected to inoculate the AnMBR in the current study with mesophilic sludge only. COD removal during the first 99 days of operation (Phase 1) was limited, averaging 57 ± 12%. Although a downward trend in permeate COD concentrations was apparent, the levels were rarely below 100 mg/L (Figure S1). Methane production was consistent with the limited COD removal and approximately half of the methane produced in the system remained dissolved in the permeate (Figure S2). The majority of the permeate COD was comprised of acetate and propionate averaging 70 ± 19 and 52 ± 18 mg/L (concentrations expressed as the compound), respectively (Figure S2). Concentrations of acetate and propionate in the bioreactor and permeate were nearly identical (data not reported) indicating negligible removal across the membranes. Inoculating the system solely with a mesophilic sludge source may have contributed to the slow startup. When using more diverse inoculum sources including biomass from a psychrophilic environment in our previous study, the startup was much faster (Smith et al., 2013). However, membrane fouling was less controlled during our previous study potentially allowing for biofilm treatment.


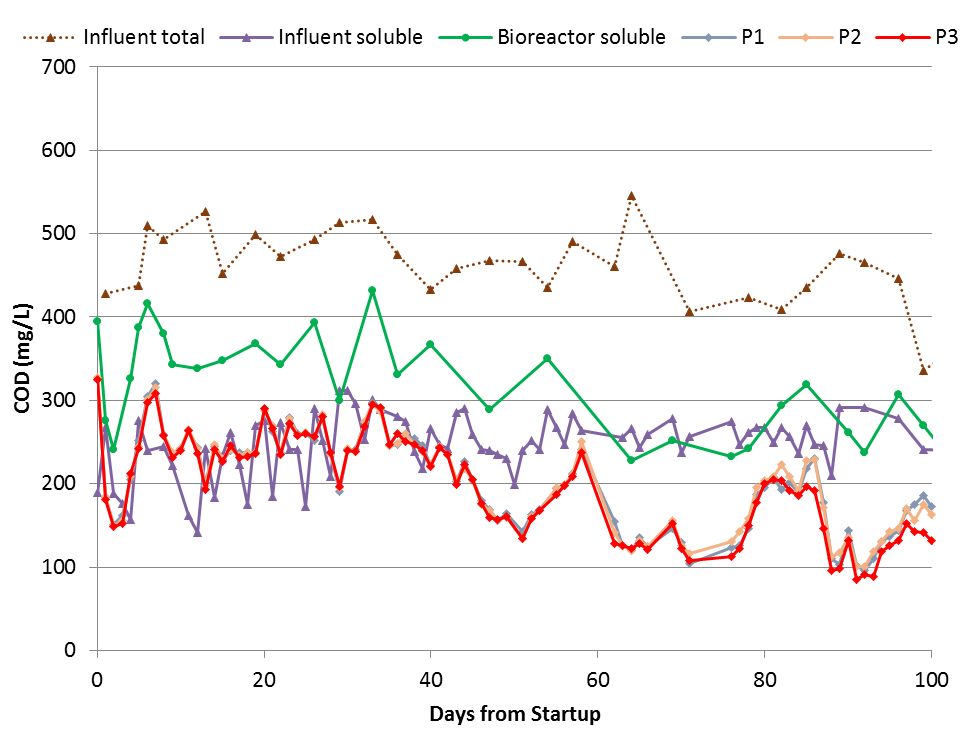


Figure S1. Influent (total and soluble), bioreactor (soluble), and permeate COD during days 1-100.


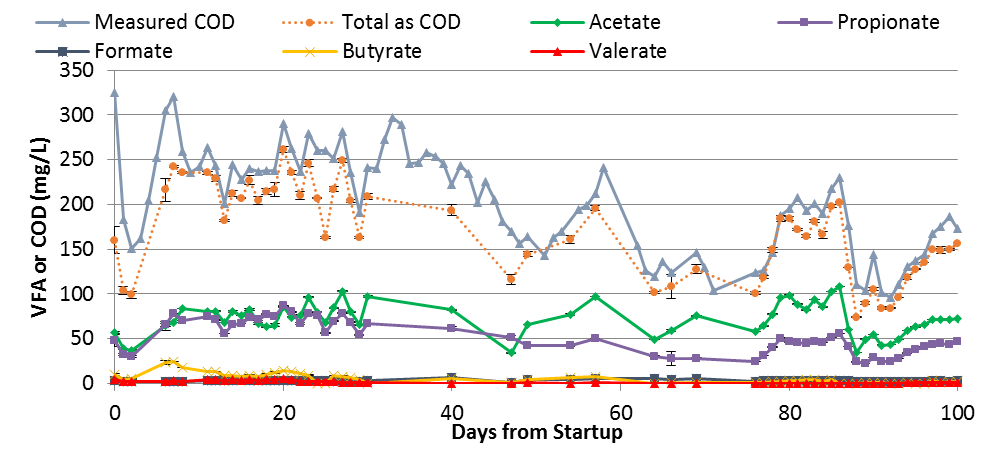


Figure S2. P1 VFA concentrations (concentrations are expressed as the actual compound, not as COD), theoretical COD contribution from measured VFAs, and measured COD during days 1-100. Total as COD is the calculated theoretical COD contribution from measured VFAs. Results for P2 and P3 were very similar (data not reported). Error bars represent standard deviations of triplicate IC injections.

Soluble COD in the bioreactor (defined as COD passing through a 0.2 µm filter, the same pore size as the membranes in the AnMBR) was consistently greater than permeate COD with an average difference of 110 ± 22 mg/L. We reported a similar observation in Smith et al. (2013). We hypothesize that this COD difference was not caused by biological mechanisms because easily biodegradable compounds such as acetate and propionate were not removed across the membrane during this time period. Further, dissolved methane concentrations in the permeate were only slightly greater than equilibrium concentrations predicted by applying Henry’s Law and the measured methane partial pressure in the biogas (averaging 1.2 times the predicted equilibrium concentration). Biological removal by the biofilm commensurate with the observed COD difference would have resulted in substantially higher methane oversaturation (discussed in main text). Based on this, we hypothesize that a physicochemical mechanism such as charge or size exclusion may have resulted in the observed COD removal across the membrane. It should be mentioned that this observed COD removal may have been influenced by differences in the physical removal capacity of the filters used to process samples and the reactor membranes. Nearly complete sulfate reduction consistently occurred in the system with influent and permeate sulfate concentrations averaging 18 ± 2.0 and 0.32 ± 0.57 mg/L, respectively. TSS and VSS concentrations initially declined but then remained stable, suggesting an initial die-off of biomass after inoculating, likely in response to the psychrophilic temperature and low OLR, which was unable to support the initial biomass concentration (Figure S4). Negligible net biomass growth occurred thereafter. The initial decline in biomass inventory corresponded to a decrease in microbial community diversity based on 16S rDNA sequencing (Figure S4), although it is unclear if the psychrophilic temperature or other extrinsic conditions were responsible for this decrease in diversity.

Our previous study reported a significantly higher COD removal of 92 ± 5%, and 21 ± 8% of the total COD removed took place across the membrane (Smith et al 2013). We further reported that increased membrane fouling resulted in greater soluble COD removal across the membrane, possibly due to biological activity in the foulant layer (Smith et al 2013). However, the improvement in COD removal when promoting membrane fouling was low, 10 ± 4 mg/L (Smith et al 2013), likely because most of the readily biodegradable substrate was already removed by the suspended biomass. We hypothesized that development of a membrane biofilm would improve effluent quality to a greater extent when the permeate contained residual biodegradable substrates (e.g., acetate or propionate) and used this strategy to attempt to improve AnMBR performance in the current study.

Figure S3. COD mass balance for days 100-138. Total COD_out_ is the summation of measured permeate COD, measured dissolved methane, measured gaseous methane, theoretical COD removal from measured sulfate reduction, and theoretical COD from measured biomass wasting.


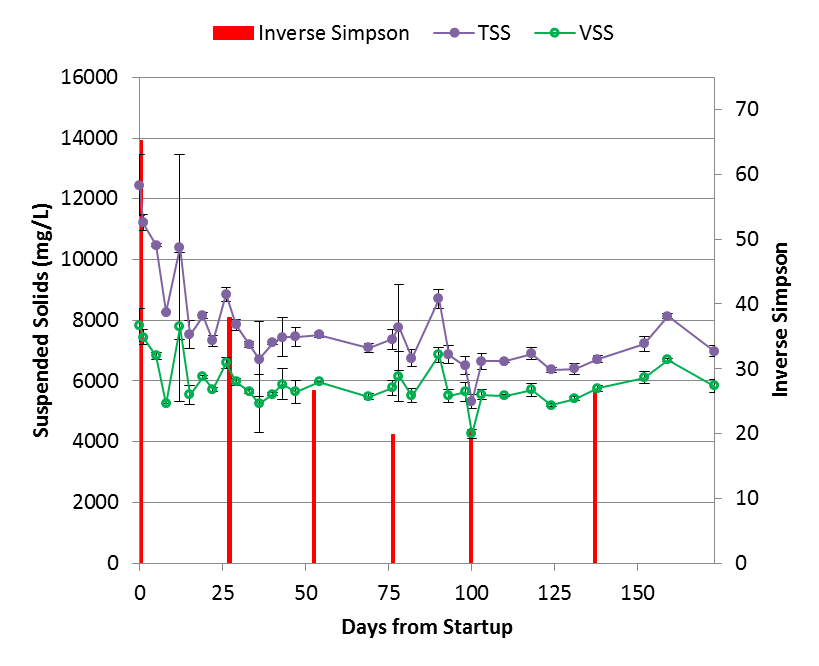


Figure S4. Total suspended solids (TSS) and volatile suspended solids (VSS) in the bioreactor during days 1-173 (primary y-axis) and inverse Simpson index in suspended biomass based on 16S rDNA sequencing (secondary y-axis). Error bars represent standard deviation of triplicate sample analysis.


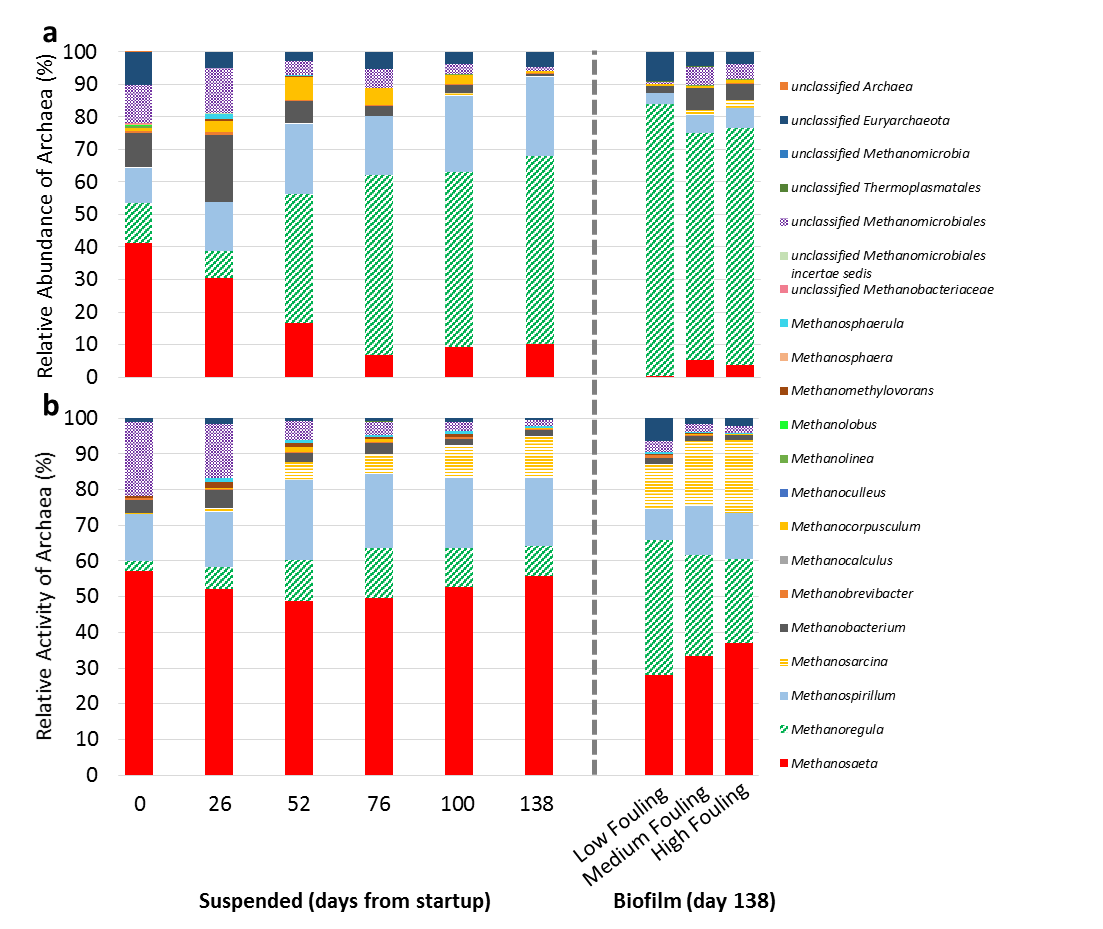


Figure S5. (a) Relative abundance of methanogens identified to the genus level based on 16S rDNA sequencing and (b) relative activity of methanogens identified to the genus level based on 16S rRNA sequencing in suspended biomass from startup to the end of Phase 2 and in biofilms at the end of Phase 2. Data are expressed as a percentage and were normalized using the total archaeal 16S rDNA sequences (a) and 16S rRNA sequences (b).


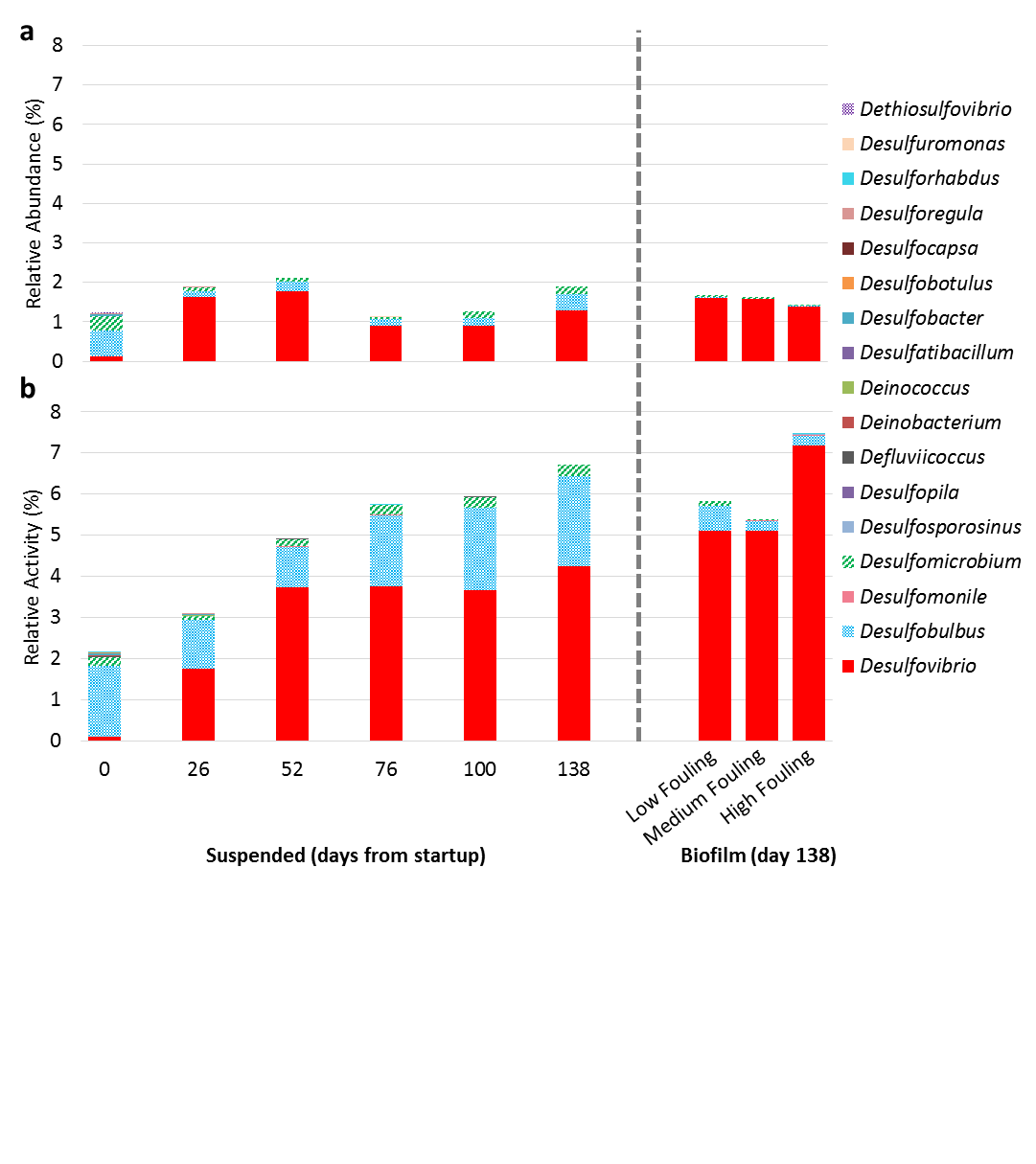


Figure S6. (a) Relative abundance of sulfate reducing bacteria identified to the genus level based on 16S rDNA sequencing and (b) relative activity of sulfate reducing bacteria identified to the genus level based on 16S rRNA sequencing in suspended biomass from startup to the end of Phase 2 and in biofilms at the end of Phase 2. Data are expressed as a percentage and were normalized using the total 16S rDNA sequences (a) and 16S rRNA sequences (b) (including archaeal and bacterial sequences). A truncated y-axis (0 to 8%) is shown to accentuate changes in abundance and activity.

Figure S7. Influent (total and soluble), bioreactor (soluble), and permeate COD during Phases 3 and 4.

**
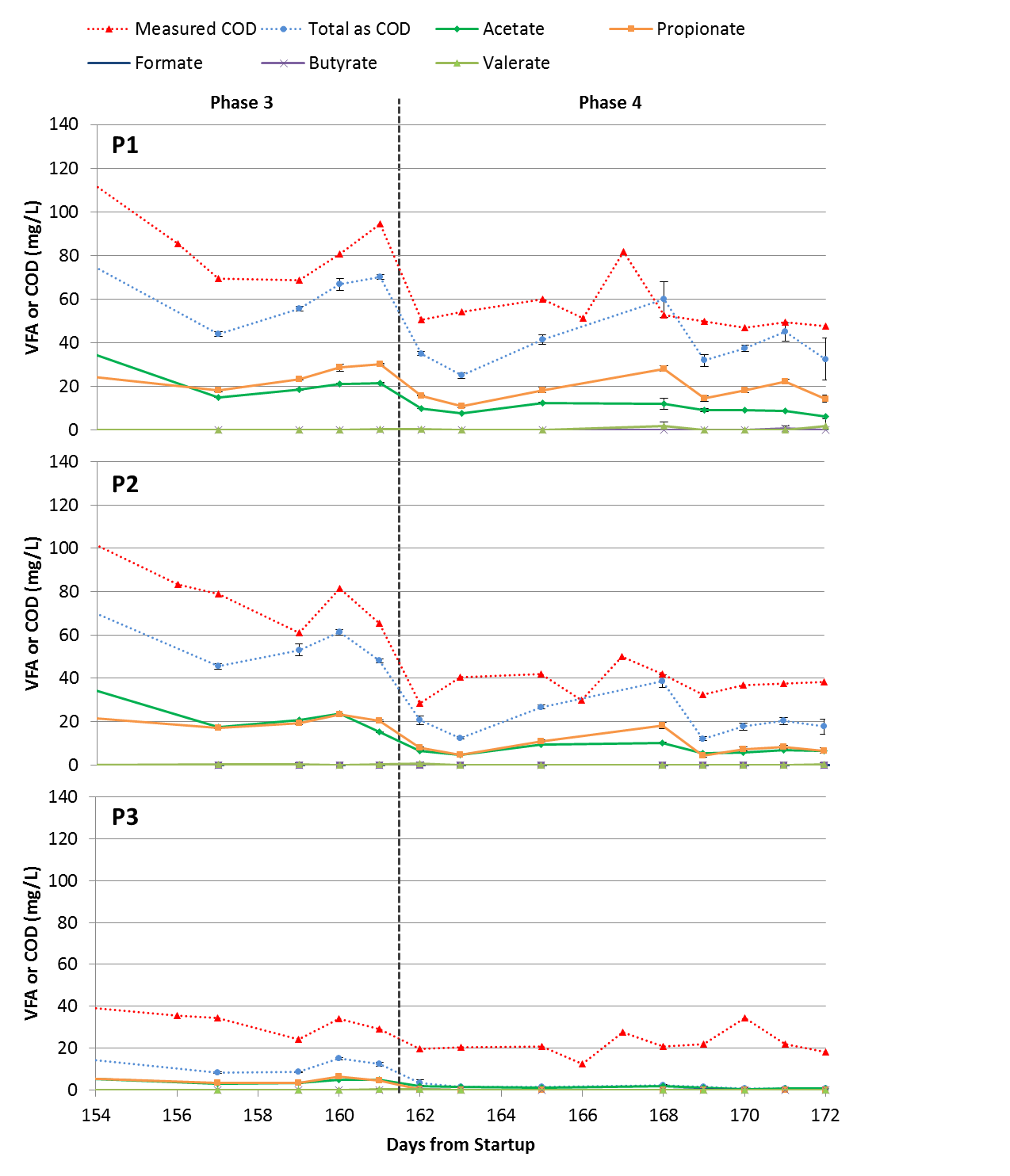
**

Figure S8. P1, P2, and P3 permeate VFA concentrations (concentrations are expressed as the actual compound, not as COD), theoretical COD contribution from measured VFAs, and measured COD during Phases 3 and 4. Total as COD is the calculated theoretical COD contribution from measured VFAs. Error bars represent standard deviations of triplicate IC injections.

**References:**

APHA (2005). *Standard Methods for the Examination of Water and Wastewater*, 21 edn. American Public Health Association: Washington, D.C.

Caporaso JG, Lauber CL, Walters WA, Berg-Lyons D, Lozupone CA, Turnbaugh PJ *et al* (2011). Global patterns of 16S rRNA diversity at a depth of millions of sequences per sample. *Proceedings of the National Academy of Sciences* **108:** 4516-4522.

Clancy TM, Smith AL, Pinto AJ, Skerlos S, Hayes KF, Raskin L (2014). Understanding the effects of methanogen inhibitors with new primers for the mcrA gene and 16S rRNA sequencing. *in preparation for Environmental Microbiology*.

He S, McMahon KD (2011). ‘*Candidatus Accumulibacter*’gene expression in response to dynamic EBPR conditions. *The ISME Journal* **5:** 329-340.

Juottonen H, Galand PE, Yrjälä K (2006). Detection of methanogenic *Archaea* in peat: comparison of PCR primers targeting the *mcrA* gene. *Research in Microbiology* **157:** 914-921.

Kozich JJ, Westcott SL, Baxter NT, Highlander SK, Schloss PD (2013). Development of a dual-index sequencing strategy and curation pipeline for analyzing amplicon sequence data on the MiSeq Illumina sequencing platform. *Applied and Environmental Microbiology* **79:** 5112-5120.

Luton PE, Wayne JM, Sharp RJ, Riley PW (2002). The *mcrA* gene as an alternative to 16S rRNA in the phylogenetic analysis of methanogen populations in landfill. *Microbiology-Sgm* **148:** 3521-3530.

Morris R, Schauer‐Gimenez A, Bhattad U, Kearney C, Struble CA, Zitomer D *et al* (2014). Methyl coenzyme M reductase (mcrA) gene abundance correlates with activity measurements of methanogenic H2/CO2‐enriched anaerobic biomass. *Microbial Biotechnology* **7:** 77-84.

Qu W, Zhou Y, Zhang Y, Lu Y, Wang X, Zhao D *et al* (2012). MFEprimer-2.0: a fast thermodynamics-based program for checking PCR primer specificity. *Nucleic Acids Research* **40:** W205-W208.

Rudd JWM, Hamilton RD, Campbell NE (1974). Measurement of microbial oxidation of methane in lake water. *Limnology and Oceanography* **19:** 519-524.

Smith AL, Skerlos SJ, Raskin L (2013). Psychrophilic anaerobic membrane bioreactor treatment of domestic wastewater. *Water Research* **47:** 1655-1665.

Sonthiphand P, Cejudo E, Schiff SL, Neufeld JD (2013). Wastewater effluent impacts ammonia-oxidizing prokaryotes of the Grand River, Canada. *Applied and Environmental Microbiology* **79:** 7454-7465.

Steinberg LM, Regan JM (2008). Phylogenetic comparison of the methanogenic communities from an acidic, oligotrophic fen and an anaerobic digester treating municipal wastewater sludge. *Applied and Environmental Microbiology* **74:** 6663-6671.

Steinberg LM, Regan JM (2009). *mcrA*-targeted real-time quantitative PCR method to examine methanogen communities. *Applied and Environmental Microbiology* **75:** 4435-4442.

Tamura K, Stecher G, Peterson D, Filipski A, Kumar S (2013). MEGA6: Molecular Evolutionary Genetics Analysis Version 6.0. *Molecular Biology and Evolution* **30:** 2725-2729.

Zeleke J, Lu S-L, Wang J-G, Huang J-X, Li B, Ogram AV *et al* (2013). Methyl coenzyme M reductase A (*mcrA*) gene-based investigation of methanogens in the mudflat sediments of Yangtze River Estuary, China. *Microbial Ecology* **66:** 257-267.
